# Neural correlates of subjective timing precision and confidence

**DOI:** 10.1038/s41598-020-59322-7

**Published:** 2020-02-20

**Authors:** Derek H. Arnold, Wiremu Hohaia, Kielan Yarrow

**Affiliations:** 1 School of Psychology, The University of Queensland, Queensland, Australia; 2 Department of Psychology, City, University of London, London, UK

**Keywords:** Sensory processing, Human behaviour

## Abstract

Humans perceptual judgments are imprecise, as repeated exposures to the same physical stimulation (e.g. audio-visual inputs separated by a constant temporal offset) can result in different decisions. Moreover, there can be marked individual differences – precise judges will repeatedly make the same decision about a given input, whereas imprecise judges will make different decisions. The causes are unclear. We examined this using audio-visual (AV) timing and confidence judgments, in conjunction with electroencephalography (EEG) and multivariate pattern classification analyses. One plausible cause of differences in timing precision is that it scales with variance in the dynamics of evoked brain activity. Another possibility is that equally reliable patterns of brain activity are evoked, but there are systematic differences that scale with precision. Trial-by-trial decoding of input timings from brain activity suggested precision differences may not result from variable dynamics. Instead, precision was associated with evoked responses that were *exaggerated* (more different from baseline) ~300 ms after initial physical stimulations. We suggest excitatory and inhibitory interactions within a winner-take-all neural code for AV timing might exaggerate responses, such that evoked response magnitudes post-stimulation scale with encoding success.

## Introduction

Humans make imprecise perceptual judgments. Repeated exposure to audio-visual inputs separated by a constant timespan can, for instance, result in variable timing judgments^1,2^. One plausible explanation would be that the dynamics of brain activity change from trial-to-trial, governing experience^3,4^. The precision of audio-visual (AV) timing judgments should then scale with variance in patterns of brain activity, evoked by identical stimulations on different trials. Another possibility is that AV stimulations evoke equally consistent patterns of activity in the brains of precise and imprecise judges of timing, but there are systematic differences that predict imprecision.

As the relationship between the dynamics of brain activity and time perception remain unclear, we decided to record the dynamics of brain activity while people performed AV temporal order judgments. We had people categorise presentations in terms of order (sound first/vision first) and confidence (low/high). We collected measures of confidence as the encoded magnitude of a timing offset should govern confidence – people should feel more confident when making decisions about big relative to small encoded timing differences^5^. Taking measures of confidence can therefore be additionally diagnostic of timing perception.

When applied to AV temporal order judgments, precision describes how rapidly a judge will switch from making predominantly sound first to predominantly vision first responses as a function of AV timing offsets, on average across many repeated trials. When we use this term, we are therefore describing the aggregate performance of a person, judging AV timing across many trials. We do not use this term to refer to decisions on a single trial.

In our experiment, trials involved the presentation of transient visual and auditory white noise signals, offset by 300, 100, or by 50 ms (with audio signals leading or lagging visual stimulations). After stimulus presentations, participants first indicated if they felt the audio or visual signal had been presented first. They then reported on their confidence in that decision. We categorised 4 types of response: high-confidence audio first, low-confidence audio first, low-confidence vision first, and high-confidence vision first (see Methods for further details).

We used multivariate pattern analyses to decode both the type of stimulation, and type of subjective timing decision, on a trial-by-trial basis from brain activity 100 ms before until 350 ms after the onset of physical stimulation. Initial analysis procedures were predetermined and preregistered (https://aspredicted.org/wi533.pdf).

## Methods

### Participants

A total of 16 people volunteered to participate, 13 male, age M 27 SD 9. One of these (DHA) was the first author. All other participants were informed they could withdraw from the study at any time without penalty. All participants provided informed consent to participate in the study. The project was approved by The University of Queensland ethics committee, and was conducted in accordance with the principles of the Declaration of Helsinki.

### Apparatus

Visual stimuli were generated using Matlab 7.5 software to drive a ViSaGe MKII Visual Stimulus Generator, and were presented on a 24″ ASUS VG248QE monitor (120 Hz refresh rate, 1920 × 1080 pixel resolution). The viewing distance was ~57 cm, controlled via a chin rest. Auditory signals were generated using a TDT Basic Psychoacoustic Workstation (Tucker-Davis Technologies) and presented diotically via loudspeakers attached to the sides of the test display. Audio presentations were synchronized with the visual display using triggers from the ViSaGe, timed to coincide with a monitor refresh.

### Stimuli

Visual stimuli consisted of circular noise patterns, with a diameter subtending 25 degrees of visual angle (dva) at the retinae, presented at 100% Michelson contrast against a black background. Individual visual noise elements subtended 0.25 dva in width and height. Visual stimuli were flashed for 1 frame (8.3 ms). Auditory stimuli consisted of 8 ms bursts of white noise, presented at a clearly supra-threshold intensity (~70 dB SPL). During blocks of trials audio presentation times were manipulated relative to visual presentation times (−300 −150 −50 +50 +150 +300 ms, with −‘ve signs indicating a visual lead) according to the method of constant stimuli. Each audio-visual offset was presented 120 times, for a total block of 720 individual trials, all completed in random order.

After each presentation participants were asked to highlight one of two response options by pressing either the left (Sound first) or right (Vision first) mouse buttons. They could then commit to this order judgment by pressing the middle mouse button. Then they were asked to report on the confidence they felt in the preceding order judgment, by adjusting the height of a bar (upward by pressing the right mouse button, downward by pressing the left mouse button). Initial bar height was randomised. If the bar was set to a height of zero, a ‘Missed Trial’ response appeared. If this response was selected, data from that trial was excluded from further analysis (this happened on a maximum of 5 from 720 trials completed by any participant). ‘Guessing’, ‘Low Confidence’, ‘High Confidence’, ‘Very High Confidence’, and ‘Certain’ response options would appear contingent on bar height, with delimit points at ¼, ½, ¾ and full possible heights. Once the desired confidence level was set, people finalised the trial by pressing the middle mouse button with the index finger of their right hands. Finishing trials with a common motor response minimised contamination by motor noise. After confidence reports there was a random delay before the next stimulation, of between 1.5 and 2.25 seconds.

Data from to-be-analysed trials (excluding ‘Missed’ trials) were sorted into four categories – high-confidence sound first, low-confidence sound first, low-confidence light first, and high-confidence light first, based on analyses of responses on each trial. For these analyses, confidence settings with bars set to ½ full possible height or less were regarded as ‘low’ confidence trials, and all greater settings as ‘high’ confidence trials.

### Analyses of behavioural data

Data for each participant described proportions of subjective audio first judgments as a function of physical audio-visual temporal offset. Data were only analysed for trials contributing to EEG data analyses, to ensure a correspondence between behavioural and brain activity analyses. Cumulative Gaussian functions were fit to these data using the psignifit toolbox version 2.5.6 for Matlab^6^, and the 50% point of fitted functions was taken as an estimate of the point of subjective AV synchrony. The slope of the fitted function at the 50% point was taken as an estimate of the *precision* of AV timing judgments (with greater slopes being indicative of greater precision).

### EEG data acquisition and pre-processing

EEG signals were acquired using a Biosemi ActiveTwo electrode system (Amsterdam, The Netherlands) from 64 AG/agCI electrodes, placed according to the extended international 10–20 system, digitized at a sample rate of 1024 Hz with 24-bit analog–digital conversion. Data were epoched offline, with a peristimulus window of −100 (before initial stimulation onset for the trial) until +350 ms (after initial stimulation). No trials were excluded from analysis based on visual inspections of data, but a large number of trials (142) was lost for one participant, due to a data recording failure. Recordings also failed for some sensors for some participants, and these were excluded from analysis (participant 1 - sensor 52, participants 4 and 5 – sensors 15 and 52, participant 6 – sensor 57). Analysed data were band-pass filtered (1 to 40 Hz) using a Butterworth filter. Blink correction was achieved using an independent component analysis (ICA) on epochs to remove ocular artifact components. All data were average referenced, and trial data were baseline corrected for each channel, relative to average voltages from −100 ms until physical stimulation onsets (0 ms).

### Decoding physical AV timing

Physical AV timing offsets were decoded via a nearest neighbour classifier with jack-knifed cross validation. Trials were classified as a −300, −100, −50, +50, +100 or as a +300 ms trial (−‘ve values indicate vision lead trials) based on similarities between measures of brain activity on a given trial and neural activation patterns elicited by each type of physical stimulation (individually for each participant) on training trials (all trials for that participant, bar the trial to be decoded on that iteration of the decoding process).

Neural activation patterns for each type of physical stimulation were estimated by averaging EEG voltages recorded at each sensor (64) and time sample (461) across all trials in the training set for the same type of physical stimulation. This created 6 signatures each defined by 29,504 features. Absolute residuals were calculated, between EEG voltages recorded for each sensor/time sample combination on the to-be-predicted trial and the 29,504 features within each of the 6 signatures. The predicted trial was classified as having matched the timing signature with which it had the smallest averaged absolute residuals. Decoding was successful when the decoded and actual input timings matched, which should happen at chance on ~17% of trials.

The statistical significance of physical input decoding for each individual was assessed via a permutation test, consisting of 2000 simulations wherein trial labels were randomly reassigned. The 2000 simulations provide a null distribution of chance decoding success rates, against which actual decoding success rates can be compared to compute a p-value.

### Decoding subjective AV timing

Subjective audio-visual timing experiences were analysed in a similar fashion to physical AV stimulation timings. Differences include that trials were decoded as a high-confidence sound first, a low-confidence sound first, a low-confidence vision first, or as a high-confidence vision first trial, all based on similarities between measures of brain activity dynamics on a given trial and signatures for each of the four types of decision calculated from all other trials. The to-be-predicted trial was classified as having matched the decisional signature with which it had the smallest averaged absolute residuals. Decoding was successful when decoded and actual decisions matched, which should happen at chance on ~25% of trials. A permutation test was conducted for each participant, as described for analyses of physical AV timing decoding. This revealed if subjective timing decisions could be decoded from brain activity at a rate above chance.

### Non-parametric cluster-based permutation tests

Individual data for these tests were averaged across all trials involving the physical input targeted for analysis. Tests were conducted using a non-parametric cluster-based permutation procedure, implemented in the Fieldtrip toolbox (www.filedtriptoolbox.org) for Matlab^7^. This allows for correlational analyses between evoked brain activity measures and behavioural outcomes, while controlling for type 1 error rate.

Three procedures were conducted. The first involved correlating individually averaged evoked potential amplitudes, at each channel/time sample combination, with individual estimates of AV timing precision. Samples with an uncorrected p-value < 0.05 were clustered based on spatio-temporal proximity, independently for positive and negative correlations. Cluster-level statistics are obtained by summing correlation statistics within each cluster, and taking the maximum to test significance against a random distribution, obtained via 1000 permutations of the original data. The second analysis was based on correlations involving individual proportions of AV timing judgments that elicited high-confidence. The third analysis involved individual estimates of inter-trial variance in evoked brain activity – standard deviations between evoked potential amplitudes, at each channel/time sample combination, correlating these features with individual estimates of AV timing precision.

## Results

All EEG data and analysis scripts for this project are available via UQeSpace https://espace.library.uq.edu.au/.

### Behavioural analyses

We estimated physical AV timing relationships coinciding with subjective synchrony (points of subjective synchrony – PSS) by fitting cumulative Gaussian distributions to individual data describing the proportion of audio first responses as a function of audio-visual offset. The average PSS estimate coincided with visual stimulations that led audio events by ~23 ms (SD = 47 ms).

**Figure 1:**
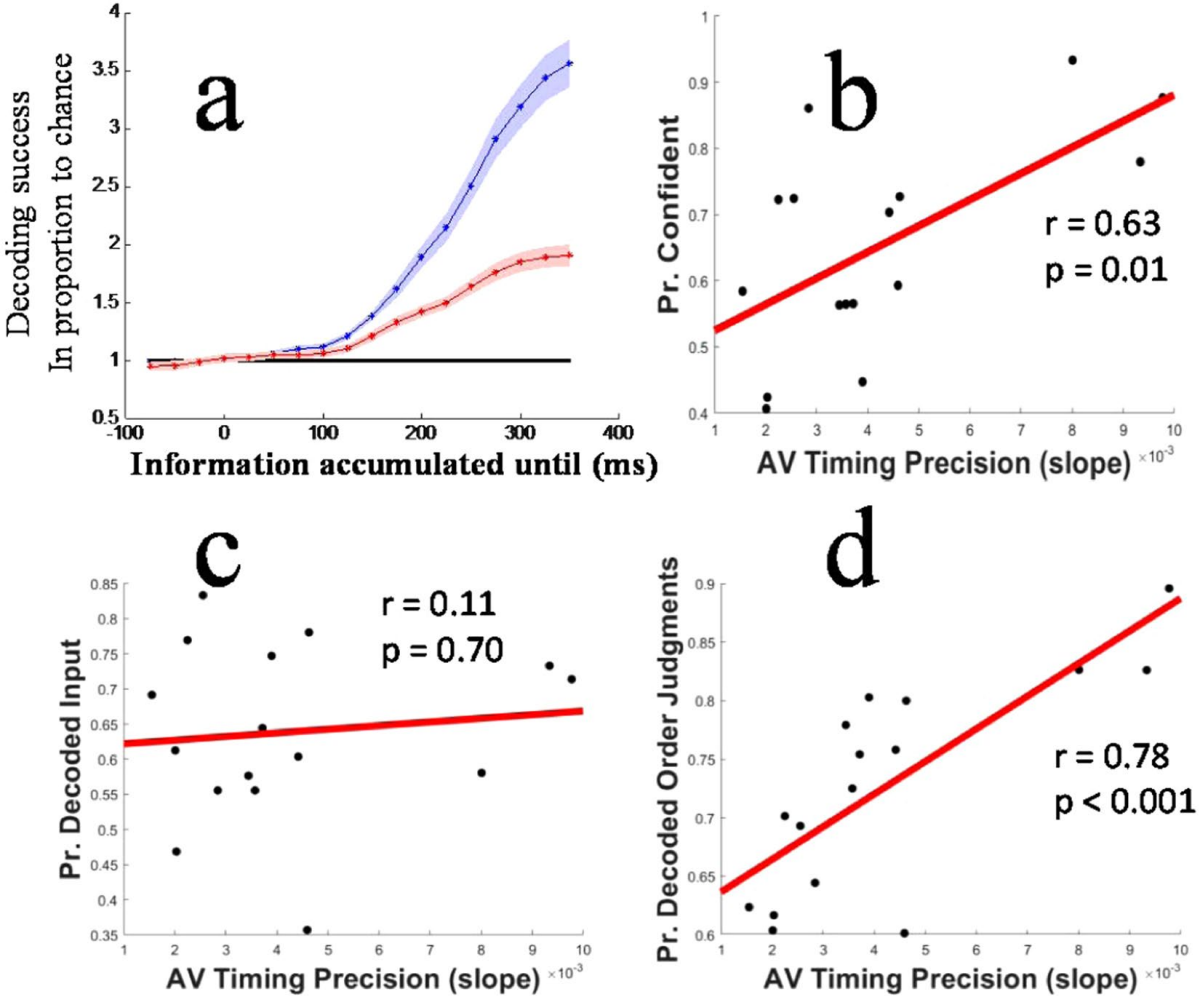
(**a**) Decoding success rates for type of physical input (blue data) and subjective timing decisions (red data) as a function of the amount of information used for decoding. Information, beginning 100 ms before stimulation, is accumulatively added to decoding processes. Data are averaged across participants and expressed in proportion to numerical chance for each process (black horizontal line). Shaded regions depict +/−1 SEM. Note that decoding for both processes begins to rise above chance in tandem, ~100 ms post stimulation. (**b**) Correlations between the proportion of trials on which participants were ‘confident’ of their timing response, and individual TOJ function slope estimates. (**c**) Correlations between the proportion of Inputs correctly decoded from brain activity and individual TOJ function slope estimates. (**d**) Correlations between the proportion of timing decisions correctly decoded from brain activity and individual TOJ function slope estimates.

We can take the slope of individual function fits, at the 50% point of the function, as estimates of the *precision* of AV timing judgments (precise judges transition more rapidly from deciding inputs are predominantly vision leading to audio leading as timing offsets change, relative to less precise judges). The average slope was 0.004 (SD = 0.003; where slope describes the additional proportion of audio first responses per ms change in relative AV timing). Previously, it has been reported that AV timing precision is *positively* associated with confidence in timing judgments^5^. Our data replicate this observation (r = 0.63, p = 0.01; see Fig. 1b).

### Input and decision decoding

The type of physical stimulation was correctly decoded from data for 59% of individual trials (SD 14), which is markedly better than chance (17%, t_14_ = 11.81, p < 0.00001). Statistical significance was assessed on an individual basis using shuffle tests, consisting of 2000 simulations wherein decoded values were randomly reassigned to different trials. The 2000 simulations provide a null distribution of chance decoding success rates, against which actual decoding success can be compared. None of the simulations resulted in a success rate that reached the actual decoding success rate for any participant (implying p < 0.005). These data show that distinct AV timings result in *reliable* differences in evoked brain activity, as decoding relies on matching evoked activity on a given trial to the *average* pattern of activity evoked by the same stimulation on other trials.

Timing decision analyses were similar. Typical activation patterns were estimated by averaging EEG voltages at each sensor and time-sample, across training sets of trials resulting in the same subjective decision. Note that these training sets encompassed trials involving *different* types of stimulation. Typical activation patterns were estimated for trials resulting in high-confidence audio first, low-confidence audio first, low-confidence vision first, and high-confidence vision first decisions. The to-be-predicted trial was classified as having matched the decision signature with which it had the smallest absolute residuals.

This decoding process resulted in correct classifications of 49% (SD 11) of trials, which is markedly better than chance (25%, t_15_ = 9.19, p < 0.00001). If we disregard confidence, the decisional classification process correctly decoded the apparent order of stimulation (vision or audio first) on 73% (SD 9) of trials. We also assessed the statistical significance of individual decisional decodings using shuffle tests, consisting of 2000 simulations wherein trial decision labels were randomly reassigned. None of the simulations for any participant resulted in a success rate that reached the actual decoding success rate for that participant. This process *did not*, however, decode information about timing decisions *independent* from the type of physical stimulation. This was evident from an additional shuffle test for each participant, wherein decision labels were only randomly reassigned between trials *involving the same type of physical stimulation*. These simulations resulted in decoding rates that were indistinguishable from chance (M 47% SD 3). All results described to this point were derived from preregistered analyses (see https://aspredicted.org/wi533.pdf). Hereafter, we describe additional follow-up analyses and results.

To further assess the success of input decoding we can fit functions to decoded data, to enable a direct comparison of these data to subjective AV timing judgments. To form decoded input functions, decoded values were transformed into binary sound/vision first data by referencing decoded values against subjective AV synchrony estimates for each individual (the 50% points of cumulative Gaussian functions fit to individual TOJ data). The slopes of functions fit to decoded input (M 0.013 SD 0.006) were steeper on average than functions fit to subjective TOJ judgments (M 0.004 SD 0.003; t15 = 6.483, p < 0.001). This shows that, on average, input decoding was more precise than the subjective timing decisions people made in relation to the decoded brain activity.

We examined how rapidly information accrued toward successful decoding by re-running our input and decision decoding processes, iteratively inputting more time samples into each analysis. We found that information for input and decision decoding became available over a similar time course, rising above chance in tandem once analyses encompassed activity ~100 ms post physical stimulation (see Fig. 1a). These data suggest that input decoding and timing decisions rely on a *common* source of information. However, these results also suggest that timing decisions are limited by an additional source of disruptive noise/information, otherwise decisions should have been decoded with equal success relative to physical input.

### Correlations between timing precision and decoding success rates

Successful decoding, of physical input timings and decisions from individual trial data, rely on *consistent* patterns of evoked brain activity on matched trials, and on these patterns being distinct for different inputs and decisions. If timing imprecision were related to variable patterns of brain activity being evoked on similar trials, input decoding success rates should have been *positively* associated with the precision of AV timing judgments. We found no evidence for this relationship (r = 0.11, p = 0.70; see Fig. 1c). A Bayesian Inference analysis regarding this correlation, implemented via SPSS v25 to estimate a JZS Bayes factor, resulted in a Bayes Factor of 4.9, which can be regarded as moderate evidence for the *null* hypothesis^8^ (that there is no relationship between subjective timing precision and input decoding success rates). When applied to correlation, Bayes factors provide an estimate of which model best describes observed data, one assuming there is no relationship between the two datasets (the null model), and one that assumes there is a linear (positive or negative) relationship. Our analysis favoured the former (null) model.

In contrast to input decoding, there was a strong positive relationship between temporal order judgment (vision or audio first) decoding success rates and the precision of AV timing judgments (r = 0.78, p < 0.0001; see Fig. 1d). A Bayesian Inference analysis regarding this correlation resulted in a Bayes Factor of 0.011, which can be regarded as very strong evidence for the alternate hypothesis (that there is a positive relationship between subjective timing precision and TOJ decoding success rates). This positive relationship suggests precise judges of AV timing may have relied *more* on the dynamics of evoked brain activity when making timing decisions than did less precise judges, as we could equally decode input timings from evoked brain activity from all participants (regardless of precision). So the decline in number of order decisions we could decode as judge precision declined cannot be due to less reliable dynamics, but may instead have been due to poor judges relying increasingly on some factor other than the dynamics of their’ own brain activity.

### Cluster tests

We reasoned that our failure to decode timing decisions, independent from the type of physical input, was a result of having included different types of stimulation in decision training sets. Due to high correlations, between types of stimulation and timing decisions, training sets were dominated by trials involving specific inputs (high-confidence audio lead training sets by −300 ms trials, etc). This could result in brain activity characteristic of specific timing decisions (irrespective of input) being masked by activity characteristic of distinct inputs (irrespective of timing decisions). To prevent this, analyses can be limited to trials involving the same type of physical stimulation. These analyses could not, however, be conducted on an individual basis due to insufficient power. Individuals completed a maximum of 120 trials per type of physical stimulation, culminating in lesser numbers of distinct timing decisions for each input.

### AV timing precision estimates and evoked brain activity correlations

**Figure 2:**
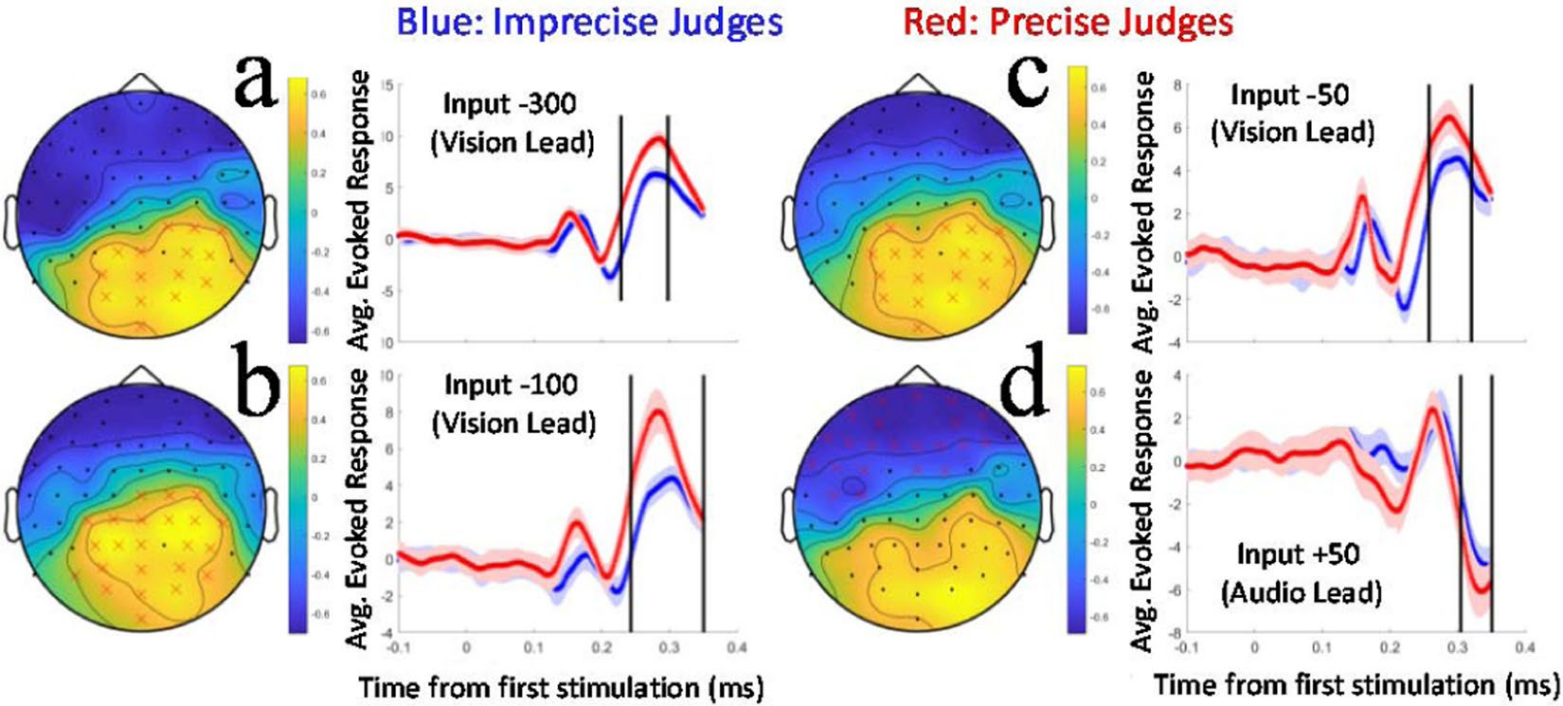
(**a**) Heat map depicting the results of a significant cluster test, based on Spearman’s correlation coefficients between average individual evoked brain activity and AV timing precision estimates. Positive correlations (yellow) indicate that evoked activity is *positively* correlated with AV timing precision. Data are from trials involving −300 ms (vision lead) stimulations. Sensors contributing to the significant clusters are indicated by red crosses. In the adjacent plot, data are split-half averaged according to timing precision (red for precise judges, blue imprecise). These data are averaged across significant cluster channels (shown in the heat map). The temporal limits of the significant cluster are indicated by the black vertical bars. (**b**) Details are as for (**a**), but data are from trials involving −100 ms (vision lead) stimulations. (**c**) Details are as for (**a**), but data are from trials involving −50 ms (vision lead) stimulations. (**d**) Details are as for (**a**), but data are from trials involving +50 ms (audio lead) stimulations, and the significant cluster describes a negative correlation.

Instead of individual trial-by-trial decoding, we conducted sequences of non-parametric cluster-based permutation tests, one for each input. For these tests, for each participant we calculate average patterns of evoked brain activity for each type of physical stimulation. Tests are then based on correlations between individual AV timing precision estimates and individual features (evoked potential amplitudes at each channel/time sample, taken from average activity patterns for each participant)^9,10^. These tests reveal if there is a robust correlation, between evoked responses and AV timing precision. We found we could reject the null hypothesis for analyses involving −300 (vision lead), −100 (vision lead), −50 (vision lead) and +50 ms (audio lead) inputs, due to significant (p < 0.05) clusters (see Fig. 2).

Readers should be mindful that the success of pattern classification analyses can be *exaggerated* by including subclasses of data within a class category^11^. Of our preceding analyses, this tendency could not have impacted input decoding, as there were no subclasses of input within those analyses (there were 6 inputs which were identical each time they were presented – so no subclasses). Decision decoding could have been impacted, as different inputs contributed to decisional signatures, which is why the current set of cluster tests were restricted to analyses of data from a signal input. Readers should also note that the split-half data appraisals we are about to describe should be regarded as illustrations (of plausible causal influences driving significant cluster tests), rather than as additional statistical analyses.

To illustrate the individual differences informing significant clusters, we split participant data based on timing precision (+/− the median precision estimate). We averaged evoked potentials across channels contributing to significant clusters, and plotted these as a function of time from initial physical stimulation (0 ms) on each type of trial. The temporal limits of significant clusters are indicated in plots via bold vertical black lines (see Fig. 2a–d). In each case, we note that for precise judges, evoked potentials are *exaggerated* ~300 ms after physical stimulation. In combination with significant cluster tests, these data show that precise and imprecise judges of AV timing have *distinct* patterns of evoked brain activity following identical physical stimulations.

**Figure 3:**
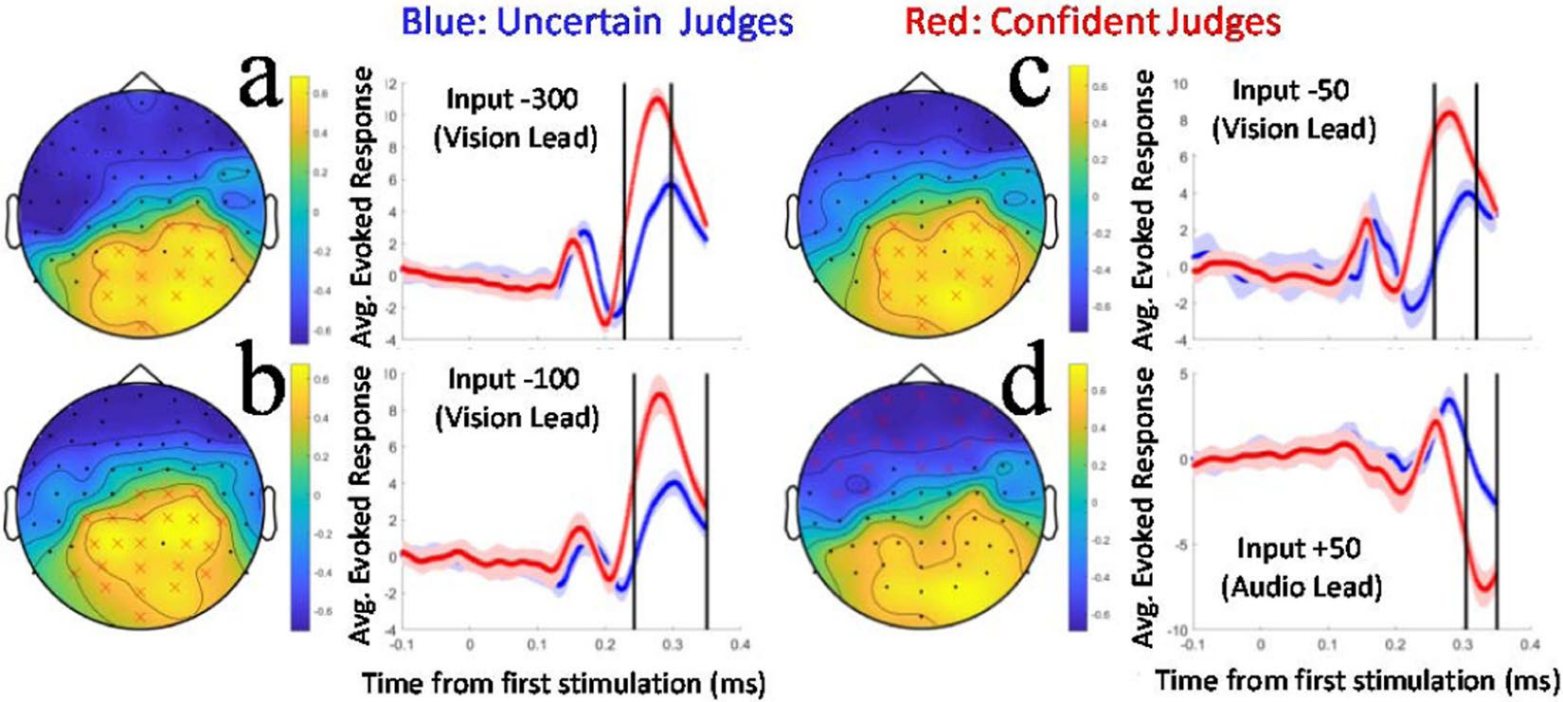
(**a**) Heat map depicting the results of a significant cluster test, based on Spearman’s correlation coefficients between average individual evoked brain activity and proportional confidence in AV timing decisions. Positive correlations (yellow) indicate that evoked activity is *positively* correlated with confidence in timing judgments. Data are from trials involving −300 ms (vision lead) stimulations. Sensors contributing to the significant clusters are indicated by red crosses. In the adjacent plot, data are split-half averaged according to confidence (red for confident judges, blue uncertain). These data are averaged across significant cluster channels (shown in the heat map). The temporal limits of the significant cluster are indicated by the black vertical bars. (**b**) Details are as for (**a**), but data are from trials involving −100 ms (vision lead) stimulations. (**c**) Details are as for (**a**), but data are from trials involving −50 ms (vision lead) stimulations. (**d**) Details are as for (**a**), but data are from trials involving +50 ms (audio lead) stimulations, and the significant cluster describes a negative correlation.

We conducted a second set of cluster-based permutation tests, based on correlations between individual proportions of timing decisions that elicited high levels of confidence, and individual EEG features. Again, we found we could reject the null hypothesis for analyses involving −300 (vision lead), −100 (vision lead), −50 (vision lead) and +50 ms (audio lead) inputs, due to significant (p < 0.05) clusters (see Fig. 3). Here too, we illustrated individual differences informing significant clusters by splitting participant data. For this analysis the split was based on the proportion of trials eliciting high-confidence (+/− the median proportion). Other details are as for our illustrations of differences relating to timing precision (see Fig. 3). We note that participants who are relatively confident when judging AV timing also have *exaggerated* evoked potentials ~300 ms after physical stimulation.

We wanted to further explore the possibility that variance between evoked activity on different trials might predict imprecision. So, we conducted a third set of cluster-based permutation tests, based on correlations between individual AV timing precision estimates and variance-based features. These features were individual standard deviations, between evoked potential amplitudes at each channel/time-sample for the same input on different trials. We could not reject the null hypothesis for any of these analyses. This provides some further evidence that AV timing precision is not predicted by variance in patterns of brain activity.

## Discussion

When discussing results, we will refer to precise and imprecise judges of AV timing. By this, we mean individuals who are more and less likely to make the same AV timing decisions about a given physical input on repeated trials. We will also refer to judges who are confident and uncertain (the opposite of confident). This refers to individuals who are more and less likely to express confidence in their timing decisions. These terms are always used to describe the average performance of a person, a judge of AV timing, across many trials. These terms do not refer to individual decisions that are right or wrong – especially given subjective timing decisions cannot be right or wrong.

Our data show that individual differences in AV timing imprecision is associated with *reliable* differences in patterns of evoked brain activity, following identical physical stimulations. Specifically, our data show that evoked responses ~300 ms after stimulation are *exaggerated* (have a greater difference from baseline) in the brains of precise, relative to imprecise, judges of AV timing (see Fig. 2). While patterns of evoked brain activity differ between precise and imprecise judges of AV timing, the distinct patterns of activity evoked in different brains seem to have been equally reliable. Trial-by-trial decoding relies on consistent, but distinct, patterns of activity being evoked by different inputs on repeated trials^12^. So equal decoding success rates, regardless of precision (see Fig. 1c), suggests that equally consistent patterns of activity were evoked in the brains of precise and imprecise judges on repeated trials with the same stimulation.

Our data also reveal *reliable* differences in evoked brain activity, between the brains of confident and uncertain judges of AV timing. These were consistent with relatively *exaggerated* evoked responses (greater differences from baseline) in the brains of confident AV timing judges, ~300 ms after physical stimulation (see Fig. 3). These data closely parallel our findings regarding precise judges of AV timing, reflecting characteristics of evoked brain activity associated with both precise AV timing judgments and confidence in those judgments.

We were able to decode AV input timings and decisions for each individual at rates that far exceeded chance. For instance, we were able to discern what decision a participant had made (sound/vision first) on ~73% of trials. Should readers be impressed by this success rate, relative to other studies? Decoding success rates in different studies will vary, depending on both the magnitude of differences in neural response patterns the study is measuring, and on measurement precision^12^, so comparing our decoding rates with studies decoding entirely different experiences would be uninformative. We are not aware of any similar set of AV timing decodings for direct comparison. All that we can say with confidence is that we were able to decode AV input timing and decisions at rates that exceeded chance. We can also note that the precision of input decoding was *superior* to the subjective timing decisions people actually made in relation to their decoded brain activity (see Results, Input Decoding).

While individual proportions of correctly decoded input did not predict AV timing precision, this was predicted by decision decoding (see Fig. 1d). These findings are not contradictory. They can be reconciled if AV timing decisions were impacted by factors untapped by our decoding processes. The robust *positive* relationship between decision decoding and AV timing precision implies that precision tended to scale with the degree to which a judge relied on the dynamics of their brain activity (as measured by our decoding processes). Less precise judges may have been increasingly influenced by factors untapped by our decoding processes, explaining the relationship between precision and decision decoding. One possible external factor is the reliability of criteria used to index brain activity on different trials. Imprecise judges may require audio signals to be encoded as leading and lagging by different magnitudes on different trials, to be regarded as leading or lagging. This could explain why they seem to have equally reliable patterns of evoked brain activity, and yet make more variable timing decisions^4^. Another possibility is that sub-cortical brain activity is influential, as this can go undetected by scalp-recordings of EEG^13^. Timing information might also be encoded via oscillatory activity^14^, which would have been smoothed by the averaging of data across trials when creating signatures for decoding.

No set of analyses can be exhaustive, so it is always possible that some different set of analyses might be more, or additionally, informative. Our study is no exception. We regard the external possibilities we have mentioned as targets for future research, and are pleased to note that other researchers can begin to investigate these matters by reanalysing the data our results are based upon – freely available, with analysis scripts, via UQeSpace (https://espace.library.uq.edu.au/). We anticipate that frequency-based analyses might be a particularly fruitful line of enquiry, given data suggesting AV synchrony perception can fluctuate at a specific duty cycle^14^. We also note that our results will need to be replicated with larger sample sizes, before we can be fully confident that they are generally representative of humans.

Here we have, however, detected reliable differences that predict AV timing precision and confidence – the magnitude of evoked responses ~300 ms after stimulation. Why might precise judges have greater evoked responses at this lag post stimulation? One possibility is that these differences are driven by excitatory and inhibitory interactions. Excitatory interactions within the modality that elicits the first cortical response on a given trial, and inhibitory interactions targeting activity evoked by the other modality, could combine to exaggerate differences from baseline. If these interactions are integral to a winner-takes-all neural code for AV timing^15,16^, evoked response magnitudes ~300 ms after stimulation could scale with encoding success, explaining the relationship we have observed.

Our research focus differs from many previous investigations of AV timing perception. We are primarily interested in the determinants of precision and confidence. Other investigations have examined where and when activity in the brain might be involved in AV timing perception, and whether different timing judgments might tap the same or different neural substrates. For instance, a broad network of cortical structures has been implicated in AV timing perception^17^, as has activity with a surprisingly short latency post stimulation (~45 ms)^18,19^. Evidence also suggests that different timing decisions rely on different substrates^20^. Our focus on the *precision* of AV timing judgments extends the scope of these investigations, and they may have more general implications, for precision in other sensory contexts.

Understanding why some people are relatively imprecise when judging AV timing might prove important for understanding and identifying debilitating psychological conditions linked to imprecise timing, such as schizophrenia and autism^21^. As yet, the reasons for some people being poor judges of AV timing are unclear. Our results suggest these people might not have more variable evoked brain dynamics, rather their brains might respond with equal reliability, but in a distinct manner. They seem to have smaller responses to input ~300 ms after stimulation. This should be further investigated, in studies similar in approach to assess the reliability of our findings, and in studies extended to encompass other combinations of sensory events, to assess the generality of the processes suggested by our data.

Overall, our data suggest that human brains are not passive recipients of timing information. Instead, they have intrinsically governed dynamics that shape precision, helping some people to be precise and confident when judging AV timing, and condemning others to be imprecise and uncertain judges.
